# Involvement and Therapeutic Potential of the GABAergic System in the Fragile X Syndrome

**DOI:** 10.1100/tsw.2010.211

**Published:** 2010-11-04

**Authors:** Inge Heulens, Charlotte D'Hulst, Sien Braat, Liesbeth Rooms, R. Frank Kooy

**Affiliations:** ^1^ Department of Medical Genetics, University of Antwerp, Belgium; ^2^ Department of Biological Sciences, Hunter College, New York, NY, USA

**Keywords:** fragile X syndrome, rational therapy, GABAergic system

## Abstract

Many drugs have been developed that are able to modulate the GABAergic system, which is involved in anxiety, depression, epilepsy, insomnia, and learning and memory. The recent observation that the GABA_A_ receptor is underexpressed in the fragile X syndrome, an inherited mental retardation disorder, therefore raised hopes for targeted therapy of the disorder. This review summarizes the lines of evidence that demonstrate a malfunction of the GABAergic system. The GABAergic system clearly emerges as an attractive target for therapy of the fragile X syndrome, and thus provides an excellent example of how genetic research can lead to unique opportunities for treatment.

## INTRODUCTION

Fragile X syndrome is the most frequent cause of inherited mental retardation affecting approximately 1 in 5000 individuals[1]. The common genetic defect is an expanding CGG repeat (dynamic mutation) in the 5' untranslated region of the fragile X mental retardation 1 (*FMR1*) gene. Elongation of this repeat above a threshold of 200 copies induces hypermethylation of the CpG island in the promoter region, leading to transcriptional silencing and, consequently, absence of the *FMR1* protein product, FMRP[2,3]. The loss of FMRP is the basic defect underlying fragile X syndrome[4]. FMRP is a RNA-binding protein that interacts with various neuronal mRNAs, and is involved in the regulation of mRNA translation, transport, and stability. At the synapses, FMRP plays a role in mRNA transport and local protein synthesis, and is therefore implicated in synaptic plasticity, a mechanism believed to underlie learning and memory[5,6]. Absence of FMRP might lead to deregulation of many neuronal mRNAs, eventually cumulating in the fragile X phenotype[7].

Genetically modified animal models, including *Fmr1* knockout mice and *dFmr1*-deficient fruit flies (*Drosophila melanogaster*), have contributed substantially to our understanding of the molecular deficits in the fragile X syndrome[8,9,10]. *Fmr1* knockout mice exhibit macro-orchidism, mild cognitive abnormalities, hyperactivity, and increased susceptibility to audiogenic seizures, features compatible with the clinical symptoms observed in fragile X patients[11,12]. In addition, dendritic spines of *Fmr1*

knockout mice are long and thin, and show an increased density, also similar to observations made in human patients[13,14,15]. Moreover, electrophysiological measurements showed a reduced long-term potentiation (LTP) in the cortex and an increased long-term depression (LTD) in the hippocampus of *Fmr1* knockout mice[16,17,18]. The *Drosophila* model shows several behavioral abnormalities, such as abnormal eclosion, courtship behavior, and circadian rhythms, and displays an abnormal neuronal morphology[19].

Despite novel insights into the pathophysiology of the disease, a specific therapy for fragile X patients is lacking. Current treatments are mostly symptom based, rather than specifically targeting the underlying neuronal defect (reviewed in [20,21]). Therefore, it is of utmost importance to further elucidate the cellular and molecular consequences of the *FMR1* mutation in order to identify new and specific targets potentially suitable for treatment of the disease. The two most prominent pathways with therapeutic potential involved in the fragile X pathogenesis involve the gamma aminobutyric acid type A ($GABA_A$) receptor and the metabotropic glutamate receptor (mGluR)[7,22]. This review will focus on the involvement of the GABAergic pathway in fragile X syndrome, as the mGluR theory has been amply reviewed most recently[23,24].

## THE $GABA_A$ RECEPTOR

$GABA_A$ receptors are the main inhibitory receptors in the brain. $GABA_A$ receptors are assembled as pentamers from 19 known different subunits ($\alpha_{1–6}$, $\beta_{1–3}$, $\gamma_{1–3}$, $\delta$, $\varepsilon$, $\theta$, $\pi$, and $\rho_{1–3}$) in a nonrandom fashion[25,26]. Different types of $GABA_A$ receptors have different physiological and pharmacological properties[27]. Most subtypes are expressed at the postsynaptic cleft, while the $\alpha_5$- and $\delta$-containing subtypes are located at extrasynaptic sites. The $\rho$ subunits are expressed only in the retina, where they assemble in a specialized set of $GABA_A$ receptors, previously known as $GABA_C$ receptors[25]. $GABA_A$ receptors are ionotropic receptors. GABA-induced stimulation occurs at the interface of the $\alpha$ and $\beta$ subunits, and results in the opening of the ion channel, resulting in an influx of $Cl^-$ ions. Consequently, the postsynaptic membrane hyperpolarizes, causing an inhibitory postsynaptic potential (IPSP) that decreases the probability of firing. These ion channels are a target for a multitude of clinically relevant drugs, including diazepam, barbiturates, and neuroactive steroids[28].

In inhibitory neurons, GABA is synthesized from glutamate by one of the two glutamic acid decarboxylase enzymes present in vertebrates, GAD65 and GAD67. The neurotransmitter is transported to the synaptic cleft in vesicular inhibitory amino acid transporters (VIAAT) and released by an action potential. In the synaptic cleft, GABA binds its receptors at the postsynaptic density. Unbound GABA is either recycled to the presynaptic terminal by one of various GABA transporters or transported to the glial cells where it is metabolized to glutamate[7,29].

In the mammalian central nervous system, 30–50% of all neurons are GABAergic[30]. $GABA_A$ receptors are detected as early as embryonic day 13 (E13)[31]. Each $GABA_A$ receptor subunit exhibits a unique regional and temporal developmental expression pattern, e.g., $\alpha_5$ and $\gamma_2$ are present in most brain regions, whereas $\alpha_2$ and $\alpha_3$ show various expression profiles in different brain regions. In adult tissue, there is still a specific regional expression pattern; however, it differs from the pattern observed in embryos, suggesting that the subunit composition and properties of the $GABA_A$ receptors are different in adult tissue compared to developing tissue.

## ALTERED EXPRESSION OF THE GABAergic SYSTEM IN FRAGILE X ANIMAL MODELS

By performing expression profiling, our research group initially observed a reduced mRNA expression level of the $\delta$ subunit of the $GABA_A$ receptor in the hippocampus and neocortex of *Fmr1* knockout mice[32]. Using real-time PCR, decreased expression of seven additional subunits of the GABA A

receptor was found in the cortex of *Fmr1* knockout mice, including the $\alpha_1$, $\alpha_3$, and $a_4$; $\beta_1$ and $\beta_2$; $\gamma_1$ and $\gamma_2$ subunits[33]. Underexpression was also found for all three subunits (*Grd*, *Rdl*, and *Lcch3*) of the *D. melanogaster* GABA receptor, suggesting that the underexpression is evolutionarily conserved. The decreased expression of the GABA receptor is directly correlated with the absence of FMRP because the reduced mRNA levels can be restored to wild-type levels by introducing transgenic constructs expressing *dFmr1* in the *dFmr1*$^{-/-}$fly. In addition, a down-regulation was also shown for several other components of the GABAergic system, such as proteins and enzymes involved in transport (*Gat1* and *Gat4*) and degradation (*Ssadh*) of GABA, and in the clustering and targeting of the $GABA_A$ receptors at the postsynaptic membrane (*Gephyrin*) in the *Fmr1* knockout mouse[34]. It is unclear whether the GABA-synthesizing enzymes *Gad1* and *Gad2*, encoding GAD67 and GAD65, respectively, are also present in reduced amounts. Our group reported decreased *Gad1* mRNA levels in the cortex of a 10-week-old *Fmr1* knockout mouse and brain of the fragile X fly model[34]. In accordance with these data, Olmos-Serrano et al. described reduced GAD65/67 protein levels in the amygdala of 3-week-old *Fmr1* knockout mice[35]. However, two other studies detected elevated GAD65/67 protein levels in the forebrain of adult *Fmr1* knockout mice[36,37]. More data are needed to resolve this issue.

In accordance with the underexpression at the mRNA level, decreased protein levels of the $GABA_A$ receptor subunits $\alpha_5$, $\beta_n$, and δ (the only subunits analyzed) were demonstrated in the hippocampus, cortex, diencephalons, and brain stem[36,38]. Most of these expression studies were performed in adult mice. Quantitative Western blotting of the forebrain of *Fmr1* knockout X mice at postnatal day 5 and 12, in comparison with adult brain, showed that multiple components of the GABAergic system were found differentially expressed at one or two developmental stages, but not in all[37]. This selective down-regulation in the first postnatal weeks may have severe consequences given the fact that there is a developmental switch from GABAergic excitation to inhibition early in the postnatal period. This brief time interval of excitatory GABAergic signaling plays a crucial role in neuronal development[39].

## FURTHER EVIDENCE FOR ABNORMALITIES OF THE GABAergic SYSTEM IN FRAGILE X SYNDROME

A dysfunction of the GABAergic system in fragile X syndrome was confirmed by many other independent groups using various techniques. Electrophysiological recordings suggested a decreased GABAergic system efficiency in *Fmr1* knockout mice that in turn may interfere with cholinergic mechanisms[40]. Neurophysiological and immunofluorescence experiments also showed that absence of FMRP is associated with apparently normal striatal glutamate transmission, but abnormal GABA transmission[41]. Recently, patch clamp recordings from subicular pyramidal cells showed that tonic GABA currents were down-regulated in neurons from *Fmr1* knockout mice, whereas no significant differences were observed in phasic currents[38]. Dictenberg et al.[42] demonstrated a reduced stimulus-induced localization of the $GABA_A$ receptor δ subunit mRNA in cultured hippocampal dendrites of neurons obtained from *Fmr1* knockout mice. This might explain the reduced protein level of the δ subunit observed in the hippocampus of *Fmr1* knockout mice[38]. Selby et al.[43] showed major defects in the neocortical and not in the hippocampal organization of GABAergic inhibitory circuits in *Fmr1* knockout mice that could be linked to the heightened sensitivity of sensory-motor gating and the increased incidence of epilepsy seen in fragile X syndrome. Reduction in the cell number and increase in the average soma area were only detected in a subpopulation of GABAergic interneurons, more specifically the parvalbumin-positive interneurons. Moreover, down-regulation of GABAergic neurons might partly explain the decrease in $GABA_A$ receptor subunits. Recently, study of the amygdala in *Fmr1* knockout mice revealed major reductions in $GABA_A$ receptor–mediated synaptic and tonic inhibition[35]. This reduction could be ascribed to the decrease in the protein levels of GAD65/67, the reduced cellular and synaptic levels of GABA, and the reduction in inhibitory synapse number they found.

## THERAPEUTIC POTENTIAL OF THE GABAergic SYSTEM IN THE FRAGILE X SYNDROME

As $GABA_A$ receptors are implicated in anxiety, depression, learning and memory, epilepsy and insomnia[44], processes also disturbed in the fragile X syndrome, we argued that the $GABA_A$ receptor might be a novel target for treatment of the fragile X syndrome[7].

The first pharmacological evidence that agonists of the $GABA_A$ receptor might be able to restore some of the deficits in the fragile X syndrome was reported recently in a fly model[45]. *Fmr1*-deficient animals die during development when raised on food containing increased levels of glutamate. Using this lethal phenotype, a chemical library of 2000 compounds was screened and nine molecules that rescued lethality were identified. Interestingly, three of them were implicated in the GABAergic pathway, including GABA itself, nipecotic acid, a GABA reuptake inhibitor, and creatinine, which is involved in a pathway that activates the $GABA_A$ receptor. Addition of each of these drugs to the food was able to restore known deficits of the fragile X flies, including *Futsch* overexpression and courtship behavior defects. Even the structural brain abnormalities in the form of morphological aberrations of the mushroom bodies, important for *Drosophila* learning and memory, could be largely rescued by addition of these drugs.

Correcting fragile X syndrome by means of GABAergic drugs will be a major challenge for future translational research. Unlike in invertebrates, food administration of GABA is not feasible in patients, as the neurotransmitter hardly crosses the blood brain barrier. However, the pharmacology of the $GABA_A$ receptor is well documented and many GABAergic drugs that are able to modulate the activity of the receptor are readily available[27]. Benzodiazepines, such as diazepam, are the best-known proven anxiolytics, but these often exhibit unwanted side effects, including sedation and dependency. However, selective $GABA_A$ receptor agonists that target defined subtypes of the $GABA_A$ receptor are currently under investigation[46]. These drugs retain the anxiolytic activity, but are devoid of the sedation liability. In that way, they might be major candidates for challenging fragile X syndrome. An entirely different type of drugs are the neuroactive steroids, such as the endogenous allopregnanolone, that are potent modulators of the $GABA_A$ receptor. A promising example of a synthetic neurosteroid is ganaxolone, which has a favorable safety profile and is now in phase II clinical trials for treatment of catamenial epilepsy[47].

## IS THE $GABA_B$ RECEPTOR ALSO INVOLVED IN THE FRAGILE X SYNDROME?

$GABA_B$ receptors are metabotropic GABA receptors. These members of the G protein-coupled superfamily are made up of a heterodimer of the membrane-spanning heptahelical proteins $GABA_{B1}$ and $GABA_{B2}$. The latter not only serves to escort $GABA_{B1}$ to the cell surface, but also links the receptor to the G protein, whereas $GABA_{B1}$ is necessary for agonist activation. $GABA_B$ receptors are found both presynaptically and postsynaptically. Upon activation, presynaptic $GABA_B$ receptors suppress neurotransmitter release through the inhibition of $Ca^{2+}$ channels or through second messenger–mediated effects[48]. Postsynaptic receptors induce a slow inhibitory postsynaptic potential by activation of G protein-coupled, inwardly rectifying, potassium channels[49]. Mice lacking functional $GABA_B$ receptors show hyperactivity and epileptic seizures, indicating that $GABA_B$ activity is crucial for normal physiological processes[50].

It has been suggested that $GABA_B$ receptors at glutamatergic terminals might serve as heteroreceptors regulating glutamate release[51]. Activation of $GABA_B$ receptors at glutamatergic spines is dependent on the spilled-over GABA from the inhibitory synapses. There is no evidence for an altered amount of $GABA_B$ receptors in fragile X syndrome[37]. However, electrophysiological experiments indicated reduced GABA concentrations in the synaptic cleft of *Fmr1* knockout mice[35]. Therefore, it is well possible that this reduced amount of GABA spill over from the inhibitory synapses results in a reduced inhibition of neurotransmitter release by the presynaptic $GABA_B$ receptors located on glutamatergic synapses (Fig. 1). Reduced inhibition at excitatory synapses may at least in part explain the increased glutamatergic signaling observed in the absence of FMRP. Such elevated signaling is believed to underlie the increased LTD observed in *Fmr1* knockout mice[17]. Thus, the $GABA_B$ receptor might provide a link with the so-called "mGluR" theory, which postulates that increased signaling though group 1 mGluR receptors is responsible for the symptoms of the fragile X syndrome, a theory that has been experimentally validated[22,52]. This link predicts that stimulating the $GABA_B$ receptor is able to diminish the excessive metabotropic glutamate signaling in the fragile X syndrome. Indeed, reduced seizures were observed in *Fmr1* knockout mice after the administration of arbaclofen, a $GABA_B$ receptor agonist[35]. In fragile X children and adults, a phase II clinical trial with arbaclofen looks promising[53]; 35% of the patients show improvement, whereby mainly the higher irritability/lower sociability and autism groups show benefit of the arbaclofen treatment.

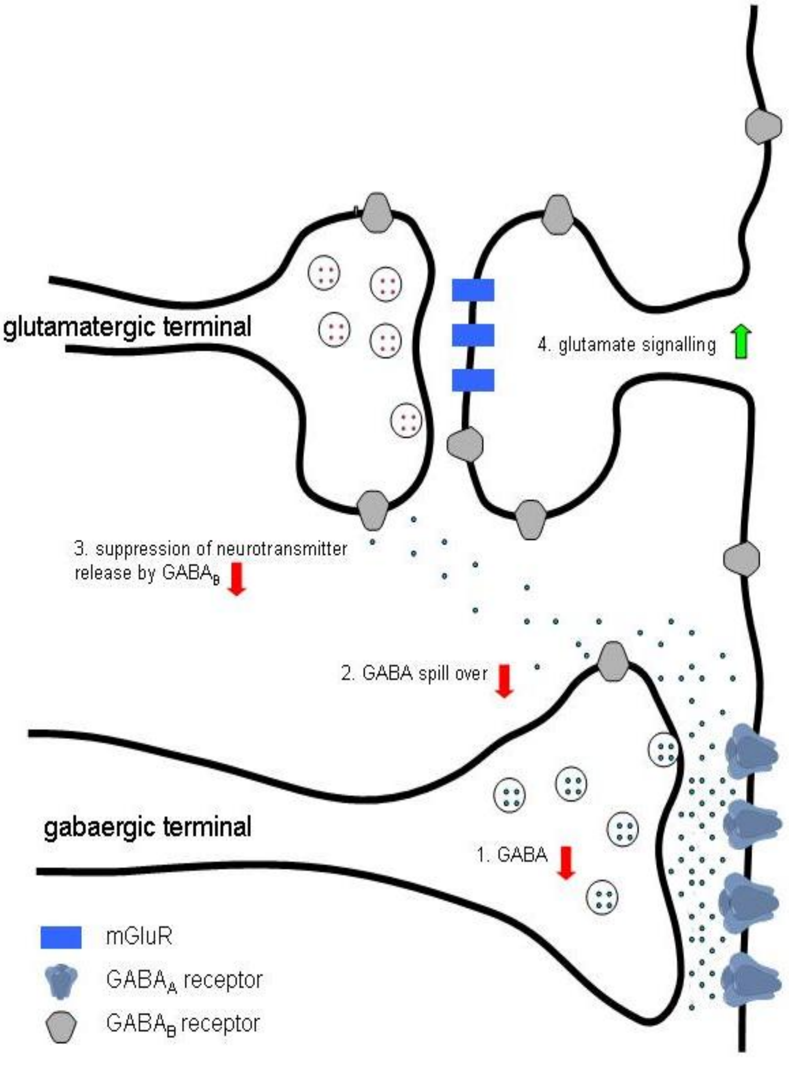

**FIGURE 1**. Hypothetical role of the $GABA_B$ receptor in fragile X syndrome. (1) GABA synthesis is reduced in the fragile X syndrome. (2) As a consequence, there might be a reduced amount of GABA spill over from the inhibitory synapses. (3) Reduced $GABA_B$ receptor stimulation results in a diminished neurotransmitter suppression by presynaptic $GABA_B$ receptors on glutamatergic neurons and may explain (part of) the excessive glutamate signaling observed in the fragile X syndrome (4).

## CONCLUSION AND PERSPECTIVE

Fragile X syndrome is an excellent example of how the identification of the causative gene in 1991[3] led to the characterization of the cellular and molecular pathways involved in the pathology of the disease, culminating in the discovery of molecular and biochemical pathways that may be targeted for rational therapy of the disorder. Several trials in fragile X patients have been initiated[20,54]. Mostly, the glutamatergic system is targeted either directly or indirectly. A complete overview of the clinical trials ongoing and planned for the near future is presented elsewhere[21].

In addition to the excitatory mGluR pathway, abnormalities of the inhibitory GABAergic system in the fragile X syndrome have now been well established. GABA is the main inhibitory neurotransmitter in the central nervous system. Its physiological function in the brain is mediated predominantly by interaction with ionotropic $GABA_A$ receptors, but also by metabotropic $GABA_B$ receptors[7]. Our research group has reported underexpression of several subunits of the $GABA_A$ receptor and of some molecules involved in GABA metabolism in the *Fmr1* knockout mouse and *Drosophila* model[32,33,34]. This finding, together with other lines of evidence, has led to the hypothesis that deficiencies in the GABAergic system may significantly contribute to the clinical symptoms of fragile X syndrome[7]. As fragile X syndrome is also the leading cause of autism, it is interesting to note that a similar down-regulation of the $GABA_A$ receptor was recently described in brains of subjects with autism[55]. The pharmacological properties of GABA receptors have been thoroughly studied, and several therapeutic agents acting on these receptors are either being evaluated in clinical trials or are already available for the treatment of neuropsychiatric disorders[7]. Promising studies in a mouse model for Down syndrome, which is shown to have an increased $GABA_A$ receptor–mediated inhibition, as opposed to the decreased GABAergic inhibition observed in *Fmr1* knockout mice, demonstrated improvement of the cognitive deficits after administration of a $GABA_A$ receptor antagonist[56]. These results suggest that $GABA_A$ receptor agonists, in addition to affecting behavior, might also be able to ameliorate cognitive functioning in fragile X patients. It is therefore encouraging to note that ganaxolone, a positive allosteric modulator of the $GABA_A$ receptor, is planned to be used for the first time in a pilot study with fragile X patients[57].

In conclusion, a dysfunction of the GABAergic system has been implicated in anxiety, depression, epilepsy, insomnia, and learning and memory, corresponding to the clinical symptoms of fragile X patients[7,58]. Targeting the GABAergic system might thus be a promising novel strategy for the treatment of fragile X syndrome.

## ACKNOWLEDGMENTS

Our research was supported through grants of the Fragile X Research Foundation (FRAXA), Institute for the Promotion of Innovation through Science and Technology in Flanders (IWT Vlaanderen), and the Belgian National Fund for Scientific Research — Flanders (FWO).
